# Incomplete echocardiographic recovery at 6 months predicts long-term sequelae after intermediate-risk pulmonary embolism. A post-hoc analysis of the Pulmonary Embolism Thrombolysis (PEITHO) trial

**DOI:** 10.1007/s00392-018-1405-1

**Published:** 2018-12-18

**Authors:** Stefano Barco, Mariaconcetta Russo, Eric Vicaut, Cecilia Becattini, Laurent Bertoletti, Jan Beyer-Westendorf, Hélène Bouvaist, Francis Couturaud, Thierry Danays, Claudia Dellas, Daniel Duerschmied, Klaus Empen, Emile Ferrari, Nazzareno Galiè, David Jiménez, Frederikus A. Klok, Maciej Kostrubiec, Matija Kozak, Christian Kupatt, Irene M. Lang, Mareike Lankeit, Nicolas Meneveau, Massimiliano Palazzini, Piotr Pruszczyk, Matteo Rugolotto, Aldo Salvi, Olivier Sanchez, Sebastian Schellong, Bozena Sobkowicz, Guy Meyer, Stavros V. Konstantinides

**Affiliations:** 1 Center for Thrombosis and Hemostasis, University Medical Center, Langenbeckstrasse 1, Mainz, Germany; 2 Department of Internal Medicine, University of Pavia, Pavia, Italy; 3 Clinical Research Unit, Assistance Publique Hôpitaux de Paris, Fernand-Widal Hospital, University Paris Diderot, Paris, France; 4 Department of Internal and Cardiovascular Medicine-Stroke Unit, University of Perugia, Perugia, Italy; 5 Department of Vascular Medicine and Therapy, Saint-Etienne University Hospital Center, Saint-Etienne, France; 6 INSERM (National Institute of Health and Medical Research) U1059, Saint-Etienne, France; 7 INSERM CIC1408, Saint-Etienne, France; 8 Department of Medicine, Division of Hematology, Thrombosis Research, University Hospital Carl Gustav Carus, Technical University Dresden, Dresden, Germany; 9 King’s Thrombosis Service, Department of Hematology, King’s College London, London, UK; 10 Cardiology Service, Michallon Hospital, Grenoble University Hospital Center, Grenoble, France; 11 Department of Internal Medicine and Pulmonology, La Cavale Blanche Hospital, CIC INSERM 1412, University of Western Brittany, E 3878 (GETBO), Brest, France; 12 Boehringer Ingelheim, Reims, France; 13 Department of Paediatric Cardiology and Intensive Care, University Medical Center Göttingen, Göttingen, Germany; 14 Heart Center, Faculty of Medicine, University of Freiburg, Freiburg, Germany; 15 Ernst Moritz Arndt Greifswald University Hospital, Greifswald, Germany; 16 Department of Cardiology, University Hospital of Nice, Nice, France; 17 Department of Experimental, Diagnostic and Specialty Medicine-DIMES, Bologna University Hospital, Bologna, Italy; 18 Department of Respiratory Diseases, Ramon y Cajal Hospital, IRYCIS, Madrid, Spain; 19 Department of Thrombosis and Hemostasis, Leiden University Medical Center, Leiden, The Netherlands; 20 Department of Internal Medicine and Cardiology, Medical University of Warsaw, Warsaw, Poland; 21 University Medical Center, Ljubljana, Slovenia; 22 Klinikum Rechts der Isar, TU Munich, and German Center for Cardiovascular Research (DZHK), Partner Site Munich Heart Alliance, Munich, Germany; 23 Department of Cardiology, Vienna General Hospital, Medical University of Vienna, Vienna, Austria; 24 Department of Internal Medicine and Cardiology, Campus Virchow Klinikum (CVK), Charité-University Medicine Berlin, Berlin, Germany; 25 Cardiology and Pulmonology Clinic, University Medical Center Göttingen, Göttingen, Germany; 26 Department of Cardiology, Equipe d’Accueil 3920, Structure Fédérative de Recherche, University Hospital Jean Minjoz, Besançon, France; 27 F-CRIN INNOVTE, St-Etienne, France; 28 Department of Cardiology, Ca´Foncello Hospital, Treviso, Italy; 29 Azienda Ospedaliero-Universitaria Ospedali Riuniti di Ancona, Ancona, Italy; 30 Université Paris Descartes, Sorbonne Paris Cité, Paris, France; 31 Pulmonology and Intensive Care Service, Georges Pompidou European Hospital, Assistance Publique Hôpitaux de Paris, Paris, France; 32 INSERM UMR S 1140, Paris, France; 33 Municipal Hospital of Dresden-Friedrichstadt, Dresden, Germany; 34 Medical University, Bialystok, Poland; 35 INSERM UMR S 970, Paris, France; 36 Democritus University of Thrace, Alexandroupolis, Greece

**Keywords:** Chronic thromboembolic pulmonary hypertension, Post-PE impairment, Pulmonary embolism, Right ventricular dysfunction, Risk stratification

## Abstract

**Introduction:**

Symptoms and functional limitation are frequently reported by survivors of acute pulmonary embolism (PE). However, current guidelines provide no specific recommendations on which patients should be followed after acute PE, when follow-up should be performed, and which tests it should include. Definition and classification of late PE sequelae are evolving, and their predictors remain to be determined.

**Methods:**

In a post hoc analysis of the Pulmonary Embolism Thrombolysis (PEITHO) trial, we focused on 219 survivors of acute intermediate-risk PE with clinical and echocardiographic follow-up 6 months after randomisation as well as over the long term (median, 3 years after acute PE). The primary outcome was a composite of (1) confirmed chronic thromboembolic pulmonary hypertension (CTEPH) or (2) ‘post-PE impairment’ (PPEI), defined by echocardiographic findings indicating an intermediate or high probability of pulmonary hypertension along with New York Heart Association functional class II–IV.

**Results:**

Confirmed CTEPH or PPEI occurred in 29 (13.2%) patients, (6 with CTEPH and 23 with PPEI). A history of chronic heart failure at baseline and incomplete or absent recovery of echocardiographic parameters at 6 months predicted CTEPH or PPEI at long-term follow-up.

**Conclusions:**

CTEPH or PPEI occurs in almost one out of seven patients after acute intermediate-risk PE. Six-month echocardiographic follow-up may be useful for timely detection of late sequelae.

## Introduction

Persisting symptoms and abnormalities of cardiorespiratory function or of echocardiographic parameters are frequently reported or detected after acute pulmonary embolism (PE). They may be accompanied by reduced exercise capacity, impaired quality of life, and overall perception of a health status which is ‘worse than before the acute PE event’ [1–7]. The frequent clinical need for caring for these patients is not met by current guidelines, which provide no specific advice on whom, when, and how to follow after acute PE [8].

Recently, the concept of post-PE impairment (PPEI) or the ‘post-PE syndrome’ was proposed, encompassing various combinations of complaints and clinical findings as well as imaging, functional or haemodynamic abnormalities, at the far end of which stands a life-threatening obstructive vasculopathy, chronic thromboembolic pulmonary hypertension (CTEPH) [1, 4, 9–11]. CTEPH is often diagnosed with delay and the identification of predictors of CTEPH after an acute PE may help to reduce this delay. The definition of PPEI continues to evolve, and it is hoped that the results of ongoing studies will help to further optimise the detection, prediction, and classification of late PE sequelae [10].

In patients with intermediate-risk PE included in the Pulmonary Embolism Thrombolysis (PEITHO) study [12], for whom long-term follow-up was available [13], we analysed cardiopulmonary symptoms and abnormal echocardiographic parameters indicating pulmonary hypertension or right ventricular (RV) dysfunction 6 months after acute PE. Our aim was to find out whether these parameters were associated with CTEPH or PPEI at long-term follow-up. We thus sought to identify predictive tools for following the course of a patient who has suffered acute PE, allowing timely detection or exclusion of late clinical and haemodynamic sequelae.

## Patients and methods

In PEITHO, a total of 1,006 patients with acute intermediate-risk PE were enrolled at 76 sites between November 2007 and July 2012 and randomised to receive tenecteplase or placebo (in addition to standard parenteral anticoagulation) [12]. Eligibility criteria included (1) an objectively confirmed diagnosis of acute PE with symptom onset 15 days or less before randomisation, (2) RV dysfunction detected on echocardiography or spiral computed tomography of the chest, and (3) a positive troponin I or T test [12]. The primary efficacy outcome was death or haemodynamic decompensation/collapse occurring within 7 days of randomisation.

An extension of the follow-up period to cover 2 years or longer was included in the third amendment of the study protocol, which was signed by 28 study sites having enrolled a total of 709 patients [13].

For the present analysis, we focused on PE survivors with available echocardiographic data at 6 months and over long-term follow-up (2 years or longer). Complete recovery of echocardiographic parameters between baseline and the 6-month visit was defined as normalisation of all echocardiographic parameters of RV dysfunction as documented by the investigators in the PEITHO case report form (Table 1). The New York Heart Association (NYHA) functional classification was used to provide an estimate of residual (or new) functional limitation during physical activity or at rest, and was assessed by the investigator team during a visit of the patient to the participating centre. The functional class prior to the acute PE event was not determined. The findings were collected in ad hoc-developed case report forms.

**Table 1:** Definition of echocardiographic recovery at 6 months and of post-PE impairment at long-term follow-up

Recovery of echocardiographic parameters between baseline and 6 months	
Echocardiographic parameters a) sPAP > 35 mmHg (vs ≤ 35 mmHg) or tricuspid systolic velocity > 2.6 m/s (vs ≤ 2.6 m/s) b) RVEDD > 30 mm (vs ≤ 30 mm) c) RVEDD/LVEDD > 0.9 (vs ≤ 0.9) d) Hypokinesia of the RV free wall	
Complete recovery	Normalisation of all the echocardiographic parameters of right ventricular dysfunction listed above
Partial recovery	Normalisation of some, but not all, echocardiographic parameters
No recovery	Normalisation of none of the parameters that were elevated or abnormal at baseline
Combined study outcome	
Confirmed diagnosis of CTEPH, or	
Post-PE impairment (PPEI), defined as a combination of the following criteria [(a) and (b) both present)]: Intermediate/high echocardiographic probability of pulmonary hypertension,* defined as estimated sPAP > 35 mm Hg, or sPAP ≤ 35 mmHg associated with at least one of the following: RVEDD > 30 mm, or RVEDD/LVEDD > 0.9 hypokinesia of the RV free wall Exertional dyspnoea of the NYHA class II, III or IV	

CTEPH, chronic thromboembolic pulmonary hypertension; LVEDD, left ventricular end diastolic dimension; NYHA, New York Heart Association; PE, pulmonary embolism; RVEDD, right ventricular end diastolic dimension; RV, right ventricular; sPAP, systolic pulmonary artery pressure

^*^The definition of echocardiographic probability of pulmonary hypertension followed the criteria recommended by current European guidelines for standardising the follow-up assessment of patients with (chronic) pulmonary hypertension, but some of the parameters and cut-off values were adapted to correspond to the data collected in the case report forms of the PEITHO trial

The outcome included a confirmed diagnosis of CTEPH or PPEI at long-term follow-up. The diagnostic workup for (suspected) CTEPH was performed at each participating site as part of standard medical care; it was not mandated by the PEITHO study protocol or its amendment concerning the extension of the follow-up period [13]. As shown in Table 1, PPEI was defined as echocardiographic findings indicating an intermediate or high probability of (chronic) pulmonary hypertension, combined with exertional dyspnoea of the New York Heart Association functional class II–IV. The definition of echocardiographic probability of pulmonary hypertension followed the criteria recommended by the current guidelines of the European Heart Association and European Respiratory Society [14], although some of the parameters and cut-off values had to be adapted to correspond to the data collected in the case report forms of the PEITHO trial (which had been defined before the pulmonary hypertension guidelines). CTEPH and PPEI cases were not independently adjudicated.

In the descriptive analysis of the patients’ baseline characteristics and study outcomes for the overall population and for each treatment arm separately, percentages were used for categorical variables, and means (standard deviation, SD) or medians (interquartile range, IQR) for continuous variables. We analysed the effects of thrombolysis on the primary outcome by means of a two-sided Chi-square test for proportions. We searched for predictors of CTEPH or PPEI (the dependent variable) among clinically selected (1) baseline clinical characteristics, and (2) parameters assessed at 6-month assessment, notably NYHA functional class and an incomplete or absent recovery of echocardiographic parameters (vs complete recovery) compared to baseline by fitting univariate and multivariable stepwise logistic regression models. Missing values of single echocardiographic parameters were considered normal if < 5% or total. SAS software 9.2 was used for data analysis.

## Results

**Fig. 1:**
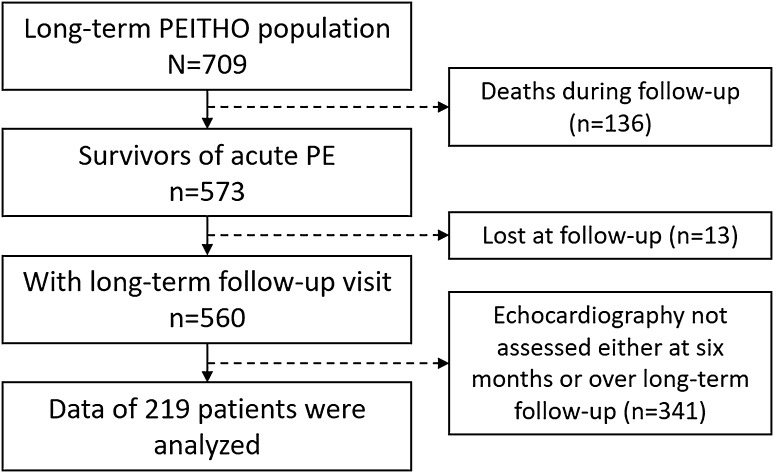
Flowchart of patient inclusion for the present analysis

Of 709 intermediate-risk patients randomised at the PEITHO sites which participated in the extension of the follow-up period, 136 patients died and 13 were lost during follow-up [13]. Among 560 survivors, echocardiography was performed in 219 patients at 6 months and over long-term follow-up (Fig. 1), of whom 112 were treated with tenecteplase and 107 with placebo. The median length of observation was 37 months (interquartile range, 27–49 months). In Table 2, the baseline parameters of survivors with echocardiographic follow-up data are compared with those without echocardiographic follow-up data.

**Table 2:** Baseline characteristics of patients with versus those without complete echocardiographic assessment

	Included (*n* = 219)	Excluded (*n* = 354)	*p*
Age (years), mean (SD)	64.8 (14.5)	64.6 (16.7)	0.90
Male sex,* n* (%)	111 (50.7)	157 (44.4)	0.14
Body weight (kg), mean, (SD)	84.0 (15.7)	83.0 (18.4)	0.50
Systolic blood pressure (mmHg), mean (SD)	133.6 (17.2)	130.5 (18.1)	0.04
Heart rate (beats/min), mean (SD)	91.3 (17.4)	93.8 (16.5)	0.08
Respiratory rate (/min), mean (SD)	21.6 (5.6)	21.4 (5.5)	0.72
Oxygen administration, * n* (%)	181 (82.6)	309 (87.3)	0.13
Chronic obstructive pulmonary disease, n (%)	7 (3.2)	15 (4.2)	0.54
Chronic heart failure, * n* (%)	9 (4.1)	15 (4.2)	0.92
Prior venous thromboembolism, * n* (%)	51 (23.3)	98 (27.7)	0.24
Active cancer, * n* (%)	8 (3.7)	16 (4.5)	0.61
Recent surgery or trauma, * n* (%)	17 (7.8)	25 (7.1)	0.75
Immobilisation, * n* (%)	25 (11.4)	30 (8.5)	0.27
Oestrogen use, * n* (%)	14 (6.4)	27 (7.6)	0.58

IQR, interquartile range; SD, standard deviation; NYHA, New York Heart Association

At long-term follow-up, 29 of 219 (13.2%) patients were diagnosed with CTEPH (*n* = 6) or fulfilled the PPEI criteria (*n* = 23). We did not detect any differences in the occurrence of CTEPH or PPEI among patients randomised to tenecteplase (14.3%) versus anticoagulation alone (12.1%) (*p* = 0.67).

Table 3 shows the unadjusted and adjusted estimates for the prediction of CTEPH or PPEI at long-term follow-up. Chronic heart failure, as reported by local study investigators, at baseline [adjusted odds ratio (OR) 7.72, 95% confidence interval (CI) 1.28–46.65] and incomplete or absent recovery of echocardiographic parameters at 6 months [adjusted OR 7.14 (95% CI 2.15–23.78)] were identified as independent predictors of CTEPH or PPEI.

**Table 3:** Factors associated with confirmed chronic thromboembolic pulmonary hypertension or post-pulmonary embolism impairment at long-term follow-up

	Unadjusted OR	95% CI	Adjusted OR	95% CI
Age ≤ 65 years	0.36	0.15–0.89	-	-
Male sex	0.47	0.21–1.08	-	-
Chronic heart failure	3.81	0.89–16.89	7.72	1.28–46.65
Active cancer	4.08	0.92–18.16	-	-
Prior venous thromboembolism	1.10	0.44–2.76	-	-
Unprovoked pulmonary embolism	0.99	0.37–2.61	-	-
Tenecteplase treatment	1.19	0.54–2.62	-	-
NYHA II, III or IV (assessed at 6 months)	3.20	1.33–7.71	-	-
Incomplete or absent recovery of echo parameters (assessed at 6 months)	4.77	1.80-12.63	7.14	2.15–23.78

*CI* confidence interval, *NYHA* New York Heart Association, *OR* odds ratio

## Discussion

We investigated the long-term clinical and haemodynamic course of 219 survivors of acute intermediate-risk PE presenting with RV dysfunction and positive cardiac biomarkers, who had been enrolled in the PEITHO trial [12]. Long-term follow-up was conducted for a median of 37 months after acute PE. This examination revealed the presence of ‘post-pulmonary embolism impairment’ or CTEPH in 13.2% of the patients. The results of our regression analysis suggest that an abnormal follow-up echocardiogram, performed ‘early’ (6 months) after acute PE, may predict an elevated risk of persistent or progressive symptoms and RV dysfunction over the long term.

Our results indicate that persistence of clinical and haemodynamic impairment is a frequent complication after intermediate-risk PE and that 6-month clinical and echocardiographic assessment may be a useful tool for predicting these late PE sequelae. Consistent with what we had previously reported [13], we did not observe any impact of systemic thrombolysis on the risk of developing CTEPH or PPEI.

The definition of PPEI is still evolving; thus far, attention has been focused on the most severe but least frequent PE sequelae, CTEPH. In this regard, existing epidemiological data are characterised by a high degree of heterogeneity. A systematic review and meta-analysis provided pooled estimates for the 2-year rate of CTEPH after PE, ranging from 0.6% among all comers to 3.2% in survivors of the acute phase [15]. Importantly, since the diagnosis and surgical treatment of CTEPH are delayed more than 1 year after the onset of symptoms [16, 17], an increased awareness for and early detection of persisting (or newly developing) pulmonary hypertension or RV dysfunction after PE might also lead to a timely diagnosis and improved management of CTEPH.

The results of the present analysis are in agreement with previous reports which suggested a relatively high incidence of echocardiographic and functional impairment after PE. For example, in a prospective cohort study of 127 patients diagnosed with ‘submassive’ PE, 17% had RV dysfunction at 6 months, 17% had functional limitation, and 8% both [2]. In another study of 78 patients with acute PE, an estimated baseline systolic pulmonary artery pressure > 50 mmHg was associated with persistence of pulmonary hypertension and signs of RV dysfunction at 1 year [18]. In the INvestigating the role oF disease monitORing in incident PE (INFORM) study, 8% of 7,068 patients with a first episode of acute PE had a medical claim for pulmonary hypertension over a 2-year period, but only half of the subjects with persisting symptoms underwent further diagnostic workup by an imaging test [19]. Finally, in the prospective Evaluation of Long-term Outcomes after Pulmonary Embolism (ELOPE) study, almost 50% of 86 patients had exercise limitation measured at cardiopulmonary exercise testing 1 year after acute PE [9]. Using a concept similar to that of our study, i.e. focusing on the importance of early assessment to predict the long-term course after acute PE, the ELOPE investigators showed that a VO_2_ peak < 80% of the predicted value on cardiopulmonary exercise testing, performed 1 month after acute PE, was significantly associated with, i.e. predicted the persistence of this abnormal finding at 1 year [9]. However, differences between the design, patient populations and outcomes in the ELOPE study and the PEITHO trial do not allow a direct comparison of the results of functional and echocardiographic assessment.

Our study has some limitations. First, we excluded patients with incomplete follow-up data; however, the comparison of baseline characteristics and 6-month findings between included and excluded patients (Table 2) suggests that selection bias is unlikely. Second, PEITHO did not include cardiopulmonary exercise or laboratory biomarker testing at follow-up, and thus the NYHA functional classification, a less standardised parameter [20], was used as the sole surrogate of functional impairment, combined with echocardiography at rest. Regarding the latter test, it needs to be mentioned that baseline and follow-up echocardiographic parameters were interpreted locally and not by a core laboratory. Third, a small overall number of patients were diagnosed with PPEI and particularly CTEPH, which led to large confidence intervals of the risk estimates limiting the ability to determine the strength of the association between the independent variables and the study outcome, and to adjust for important additional covariates. Finally, we cannot exclude the possibility that some patients may already have had CTEPH at the time of inclusion in PEITHO [21]. This represents a limitation of most existing studies in the field, as they were not designed to systematically search for pre-existing CTEPH using standardised criteria. Therefore, our findings might partly reflect a chronic condition present prior to the acute PE event, including pre-existing CTEPH or pulmonary hypertension of other cause(s), which may have contributed to the identification of ‘chronic heart failure’ as a significant predictor of outcome.

In light of the above limitations, and to the fact that no standardised CTEPH diagnosis protocol was mandated by the PEITHO trial protocol, no firm conclusions can be drawn from our analysis regarding the possible efficacy of systemic thrombolysis for prevention of late PE sequelae. Moreover, neither the PEITHO trial nor the present analysis was designed to directly address the question on which proportion of the patients with PPEI may ultimately progress to CTEPH, and at what rate this may happen.

In conclusion, we found that chronic thromboembolic pulmonary hypertension or the combination of exertional dyspnoea with persistent or progressing right ventricular dysfunction (termed post-pulmonary embolism impairment) occurs in almost one out of seven patients after acute intermediate-risk pulmonary embolism. Our results, which suggest that 6-month echocardiographic follow-up may be useful in detecting or predicting late sequelae of pulmonary embolism, must be confirmed by future, appropriately designed studies.
